# Impact of adiposity on muscle function and clinical events among elders with dynapenia, presarcopenia and sarcopenia: a community-based cross-sectional study

**DOI:** 10.18632/aging.202581

**Published:** 2021-02-26

**Authors:** Tung-Wei Kao, Tao-Chun Peng, Wei-Liang Chen, Der-Sheng Han, Chi-Ling Chen, Wei-Shiung Yang

**Affiliations:** 1 Division of Geriatric Medicine, Department of Family and Community Medicine, Tri-Service General Hospital, National Defense Medical Center, Taipei, Taiwan; 2 Graduate Institute of Clinical Medicine, College of Medicine, National Taiwan University, Taipei, Taiwan; 3 Department of Physical Medicine and Rehabilitation, National Taiwan University Hospital BeiHu Branch, Taipei, Taiwan; 4 Department of Internal Medicine, National Taiwan University Hospital, Taipei, Taiwan; 5 Center for Obesity, Life Style and Metabolic Surgery, National Taiwan University Hospital, Taipei, Taiwan

**Keywords:** dynapenia, obesity, fall, metabolic syndrome, fat to muscle ratio

## Abstract

Introduction: Low muscle function determined unfavorable clinical outcome than low muscle mass; nevertheless, comparison of detrimental parameters among dynapenia, presarcopenia and sarcopenia was sparse. We hypothesized that adiposity is implicated in low muscle function related adverse events.

Methods: We recruited community elders to measure handgrip strength and walking speed. Using bioelectronics impedance analyzer to examine body compositions. The faller is indicated of having a fall event in the past one year. Associations of different obesity parameters, metabolic syndrome (MetS) and fall among the groups were analyzed.

Results: Among 765 participants, the dynapenia group had higher metabolic profiles, body fat percentage (BFP), waist circumference, and fat to muscle ratio (FMR) than the other groups, whereas the presarcopenia subjects had the lowest obesity parameters. The fallers tended to have poorer muscle function than non-fallers (p<0.001). The dynapenia individuals had the highest risk for MetS (odds ratio [OR]= 5.79; 95% confidence interval [CI]= 2.45-13.73), and the highest fall risk (OR= 3.11; 95% CI=1.41-6.87). Among obesity parameters, FMR had better diagnostic performance to estimate low muscle function, followed by BFP.

Conclusion: Dynapenia individual had higher risk of obese-related adverse events. Increased adiposity irrespective of muscle mass is relevant to reduced muscle function among elders.

## INTRODUCTION

The impact of muscle mass or muscle strength on functional disability has attracted high levels of attention in the medical community [1–3]. In 1989, Rosenberg described the concept of aging-related skeletal muscle mass decline [4]. In addition, some studies have addressed the influence of muscle strength on functional loss and clinical events in recent years [5–7]. Muscle mass and muscle strength have been discussed together since presarcopenia and sarcopenia were defined in 2010 by the European Working Group on Sarcopenia in Older People (EWGSOP) [8]. Most researchers have recognized that both muscle quantity and quality might have equal contributions to clinical adverse events. Older adults with sarcopenia, or even more with severe sarcopenia, tended to have a higher risk of morbidity and mortality [9].

Beyond the concepts of presarcopenia and sarcopenia, Clark and colleagues had innovatively described the concept of low muscle strength without low muscle mass as dynapenia [10]. In line with this definition, a meta-analysis from 42 longitudinal studies explored the idea that low muscle strength tended to play a more critical role in functional decline and poor health outcomes than low muscle mass among older adults [11]. In contrast, the other study found that both low muscle mass and low muscle strength played a synergistic effect in increasing the risk of losing physical independence [12]. However, studies comparing the impact on clinical adverse events among dynapenia, presarcopenia and sarcopenia in older individuals are scarce [13, 14].

Along with skeletal muscle loss, increased fat mass is another issue that has been extensively investigated in recent years. In fact, high percentage of older adults who suffer from sarcopenia had obesity [15]. Some reports revealed that obesity appeared in older adults with dynapenia [16, 17]. However, different body fat profiles of older people characterized as having presarcopenia, dynapenia or sarcopenia have not been compared. The fat and muscle effects on metabolic risk and clinical adverse event such as fall among people with dynapenia, presarcopenia and sarcopenia have rarely been explored as well. Fall risk estimation is a major and common outcome in geriatric syndrome leading to further functional decline. Nevertheless, different parameters and cutoff values composed of different definitions of sarcopenia and dynapenia to examine this issue. We tried to further examine whether muscle function is a major determinant factor more than muscle mass on fall risk. We hypothesized that low muscle function increased the risk of adverse clinical events; whereas adiposity is implicated in its association. The aim of this study was to examine the relationships of different measurements of body fat and the related risks of metabolic syndrome and fall event in community-dwelling older adults who were at different levels of dynapenia and sarcopenia. In addition, we explored which adiposity parameter is the most relevant determinant for reduced muscle function.

## RESULTS

### Baseline characteristics

**Figure 1:**
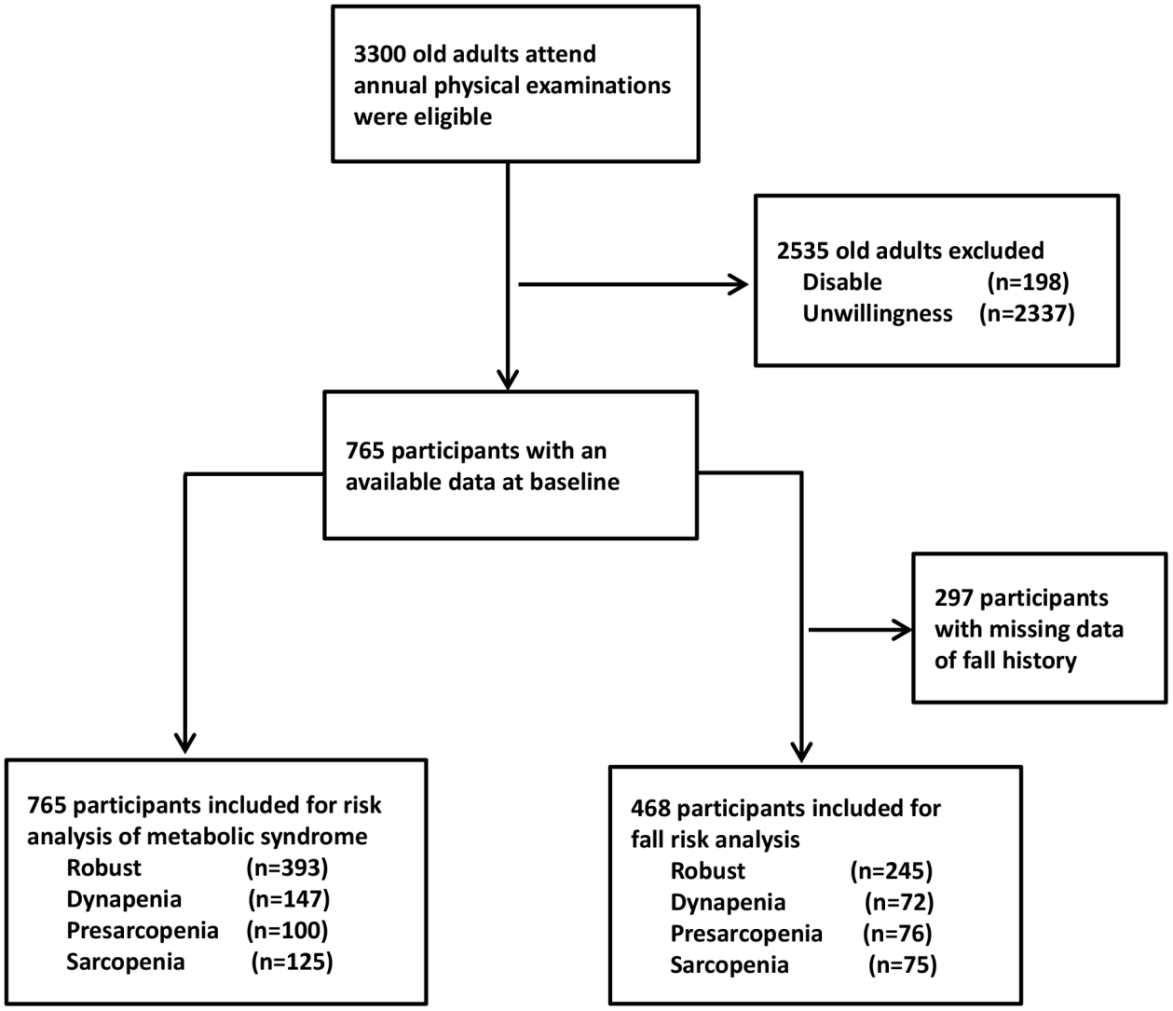
**Flowchart of the sample selection in the study.**

There were 3300 older adults who visited our medical center in Taipei City for an annual routine check-up were eligible. After screening for exclusion criteria, there were 198 old adults unable to perform physical exercise due to disability (n=198), and 2337 old adults with unwillingness to join this study (n=2337); hence, there were 765 old adults entered our study. In addition, there were 297 participants with incomplete data of fall history (n=297); therefore, 468 participants contributed data to the fall risk analysis (Figure 1). They were categorized into 4 groups: robust, dynapenia, presarcopenia and sarcopenia based on their muscle mass and function as in Table 1. In general, the participants with dynapenia tended to have the highest BMI, waist circumference, fat mass, BFP and FMR as well as the highest rate of metabolic syndrome among the four groups, whereas presarcopenia individuals had the lowest obesity profile and rate of metabolic syndrome among the four groups (Table 1). In terms of certain body compositions, such as BMI, waist circumference and SMI, the robust and dynapenia can be categorized as a group (normal muscle mass group); whereas the presarcopenia and sarcopenia can be grouped together (low muscle mass group). In contrast, for BFP and FMR, the sarcopenia group was similar to the dyapenia group (low muscle strength group), while the presarcopenia was similar to the robust (normal muscle strength group).

**Table 1:** Characteristics of the participants categorized by muscle mass and muscle strength.

	**Normal muscle mass**		**Low muscle mass**		**p-value**
	**normal muscle strength**	**low muscle strength**	**normal muscle strength**	**low muscle strength**	
	**Robust n=393**	**Dynapenia n=147**	**Presarcopenia n=100**	**Sarcopenia n=125**	
Continuous variables ^*^					
Age (yrs)	71.08±6.18	74.88±7.83	73.23±7.29	78.25±7.85	<0.001
BMI (kg/m^2^)	25.19±3.03	26.45±3.49	21.67±2.36	22.2±2.83	<0.001
Waist circumference (cm)	82.57±9.53	84.85±9.64	74.44±7.99	77.05±8.96	<0.001
SBP (mmHg)	131.85±15.68	133.69±15.57	129.44±17.37	130.42±16.81	0.19
Grip strength (kg)	29.6±8.48	19.51±7.2	26.67±6.81	16.73±6.04	<0.001
Gait speed (m/s)	1.31±0.31	0.91±0.34	1.32±0.26	0.97±0.32	<0.001
Physical activity(kcal/week)	8913.8±2830.2	8252.1±2998.8	9305.9±3330.2	8433.7±2551.7	0.013
SMI (kg/m^2^)	7.1±0.91	6.95±0.93	5.71±1.09	5.39±1.21	<0.001
Fat mass(kg)	20.18±6.07	22.4±6.96	15.51±4.23	17.29±5.42	<0.001
Body fat (%)	30.6±7.24	33.95±7.74	29.11±6.98	32.29±7.45	<0.001
Fat/muscle ratio	0.85±0.3	1.0±0.34	0.81±0.28	0.95±0.31	<0.001
HDL(mg/dL)	54.59±14.2	54.78±12.7	61.27±15.17	57.8±14.73	<0.001
LDL(mg/dL)	108.81±28.59	106.67±29.18	117.01±32.31	105.59±27.91	0.022
Uric acid (mg/dL)	5.77±1.36	5.54±1.34	5.33±1.3	5.5±1.33	0.017
TG (mg/dL)	118.79±62.36	109.89±46.04	98.96±38.86	107.58±49.09	0.008
Fasting Sugar(mg/dL)	102.09±19.94	104.46±22.52	98.05±16.02	101.75±25.42	0.151
Categorical variables ^†^					
Male	190(48.3)	53(36.1)	42(42)	40(32)	0.003
Smoker	16(4.1)	2(1.4)	0	0	0.788
Alcohol consumption ≥4 times/month	41(10.4)	6(4.1)	4(4)	1(0.8)	0.724
Metabolic syndrome	107(27.2)	45(30.6)	7(7)	23(18.4)	<0.001
Hypertension	148(37.7)	76(51.7)	23(23)	55(44)	<0.001
Diabetes	49(12.5)	33(22.4)	10(10)	19(15.2)	0.015
Stroke	5(1.3)	5(3.4)	1(1)	6(4.8)	0.069
CAD	21(5.3)	19(12.9)	6(6)	11(8.8)	0.001
Arthritis	83(21.1)	44(29.9)	19(19)	33(26.4)	0.088
Osteoporosis	60(15.3)	34(23.1)	19(19)	29(23.2)	0.820
Anti-psychotics	5(1.3)	3(2)	1(1)	6(4.8)	0.016
Sedative agents	63(16)	38(25.9)	24(24)	30(24)	0.027

^*^Values in the continuous variables were expressed as mean ± standard deviation unless otherwise specified. BMI, body mass index; CAD, coronary artery disease; HDL, high-density lipoprotein; LDL, low-density lipoprotein; SBP, systolic blood pressure; SMI, skeletal muscle mass index; TG, triglyceride ^†^ Values in the categorical variables were expressed as number (percent).

### Metabolic syndrome in dynapenia, presarcopenia and sarcopenia

The risks of metabolic syndrome were analyzed using the presarcopenic group as the reference. The dynapenic older adults had the highest rate of metabolic syndrome than the participants in robust and sarcopenia groups (Odds Ratio [OR]= 6.12, 95% confidence interval [CI]= 2.62-14.33; OR= 5.24, 95% CI= 2.35-11.72; and OR= 3.21, 95% CI=1.30-7.94, respectively). This finding was consistent even after performing adjustments with multiple covariates in further statistical analyses (Table 2).

**Table 2:** Risk of metabolic syndrome in the participants with dynapenia, presarcopenia and sarcopenia.

	**Robust n=393**		**Dynapenia n=147**		**Presarcopenia n=100**	**Sarcopenia n=125**	
Models*	OR (95% CI)	p-value	OR (95% CI)	p-value		OR (95% CI)	p-value
Model 1	5.24 (2.35-11.72)	<0.001	6.12 (2.62-14.33)	<0.001	1.00(Ref.)	3.21 (1.30-7.94)	0.012
Model 2	4.79 (2.13-10.79)	<0.001	5.79 (2.45-13.73)	<0.001	1.00(Ref.)	2.95 (1.18-7.36)	0.021

^*^Adjusted covariates: Model 1= age, gender; Model 2=Model 1+ health behaviors (smoking and alcohol consumption), physical activities, uric acid, stroke and coronary artery disease; CI, confidence interval; OR, odds ratio; Ref, reference.

### Fall risk in subgroup analysis

Among these older adults, 468 participants with complete records of fall history were used for the subgroup fall risk analysis. The participants with fall history (*n*= 60) tended to have lower grip strength and slower gait speed than the non-faller individuals. Gender, metabolic syndrome, body compositions such as SMI, fat mass, BFP and FMR revealed no significant differences between the fallers and non-fallers (Table 3). Fall risk was significantly associated with grip strength and gait speed in a negative manner (OR= 0.92, 95% CI =0.88-0.97; and OR= 0.30, 95% CI= 0.11-0.78, respectively) by logistic regression analyses with adjustment with age, gender, health behaviors, metabolic syndrome, physical activities, osteoporosis, arthritis, and use of anti-psychotics or sedative agents. In contrast, no significant relationship was observed between fall risk and all kinds of obesity related body compositions.

**Table 3:** The comparison between the participants with or without fall history.

	**Faller n=60**	**Non-faller n=408**	**p-value**
Male, n (%)	23 (38.3)	175 (42.8)	0.505
Handgrip strength (kg)	22.03±9.28	26.48±9.27	0.001
Low handgrip strength, n (%)	26 (43.3)	83 (20.3)	<0.001
Gait speed (m/s)	1.0±0.38	1.19±0.36	<0.001
Slow gait speed, n (%)	21 (35)	53 (12.9)	<0.001
BMI (kg/m^2^)	24.71± 4.35	24.40± 3.43	0.534
Waist circumference (cm)	83.54± 11.25	81.30± 9.72	0.110
SMI (kg/m^2^)	6.19±1.84	6.53±1.19	0.062
Fat mass (kg)	19.88±7.26	19.52±6.32	0.690
Body fat (%)	32.35±6.91	31.61±7.54	0.484
FMR	0.93±0.32	0.89±0.32	0.496
Metabolic syndrome, n (%)	12 (20)	98 (24)	0.484

Values were expressed as mean ± standard deviation or number (percent). BMI, body mass index; SMI, skeletal muscle mass index; FMR, fat to muscle ratio.

The participants with dynapenia had the highest fall events than the other groups (17 individuals, 23.6%), whereas the subjects with presarcopenia had the lowest fall events (6 individuals, 7.9%). In fact, the fall rate of robust was similar to that of presarcopenia, while the fall rate of sacrcopenia was similar to that of dynapenia (Table 4). The OR of fall events among the participants with dynapenia, presarcopenia and sarcopenia were 3.21 (95% CI= 1.54-6.64), 0.92 (95% CI= 0.35-2.39), and 2.74 (95% CI= 1.24-6.05), respectively. The fall risk was still higher in the dynapenia group than in the sarcopenia group after multiple covariates were adjusted in further analyses (OR= 3.11, 95% CI= 1.41-6.87; and OR= 2.80, 95% CI=1.18-6.69, respectively) (Table 4). These results suggest that muscle strength is more relevant than body compositions, including muscle mass in view of fall risk.

**Table 4:** Associations of fall risk between the participants with dynapenia, presarcopenia and sarcopenia.

	**Robust n=245**	**Dynapenia n=72**		**Presarcopenia n=76**		**Sarcopenia n=75**	**p-value**
Fall, n(%)	20(8.1)	17(23.6)		6(7.9)		17(22.7)	<0.001
Models*		OR (95% CI)	p-value	OR (95% CI)	p-value	OR (95% CI)	p-value
Model 1	1.00(Ref.)	3.21 (1.54-6.64)	0.002	0.92 (0.35-2.39)	0.859	2.74 (1.24-6.05)	0.013
Model 2	1.00(Ref.)	3.11 (1.41-6.87)	0.005	0.85 (0.31-2.29)	0.744	2.80 (1.18-6.69)	0.020

^*^Adjusted covariates: Model 1= age, gender; Model 2=Model 1+ health behaviors (smoking and alcohol consumption), metabolic syndrome, physical activities, osteoporosis, arthritis, use of anti-psychotic agents and sedative agents. CI, confidence interval; OR, odds ratio; Ref, reference.

### Diagnostic performance of adiposity on low muscle function

Among various obesity parameters, BMI, BFP and FMR had significant diagnostic performance on low handgrip strength and low gait speed. However, WC and fat mass had significant diagnostic performance only on low gait speed but not on low handgrip strength (Table 5). In estimating the risk for low handgrip strength, FMR had the best diagnostic performance among these obese parameters (AUC= 0.617, 95% CI= 0.573-0.661). In the prediction of low gait speed, the best diagnostic performance was still FMR (AUC= 0.622, 95% CI =0.563-0.682) than by the other obese parameters. Although BFP was the second best, its performances were close to those of FMR (Table 5, Supplementary Figure 1). These data suggest obesity irrespective of muscle mass (dynapenic or sarcopenic obesity) is relevant to reduced muscle function, especially for lower extremity in the elderly.

**Table 5:** Diagnostic performance of different obese parameters on low handgrip strength and low gait speed.

	**Low handgrip strength**		**Low gait speed**	
	**AUC (95%CI)**	**p-value**	**AUC (95%CI)**	**p-value**
BMI (kg/m2)	0.551 (0.504-0.598)	0.029	0.567 (0.508-0.627)	0.024
WC (cm)	0.523 (0.476-0.569)	0.328	0.587 (0.529-0.645)	0.004
Fat mass (kg)	0.501 (0.455-0.546)	0.977	0.567 (0.507-0.627)	0.026
Body fat percentage (%)	0.601 (0.556-0.646)	<0.001	0.613 (0.553-0.673)	<0.001
Fat / muscle ratio	0.617 (0.573-0.661)	<0.001	0.622 (0.563-0.682)	<0.001

AUC, area under the curve; BMI, body mass index; CI, confidence interval; WC, waist circumference.

## DISCUSSION

In this study, we found that participants with dynapenia had the highest obesity profile among all groups, whereas the presarcopenia group had the lowest obesity characteristics. Previously, it was proposed that visceral and intermuscular adiposity increased concomitantly with skeletal muscle mass reduction during the aging process [18, 19]. In this study, it is evident that increased levels of fat mass were associated with dynapenia, a status defined solely by muscle function loss, without muscle mass reduction. Therefore, the loss of muscle function was associated with multiple obesity parameters, such as increased BMI, WC, fat mass, BFP, and especially FMR. In contrast, older adults with only loss of muscle mass, presarcopenia, tended to have good muscle function and higher physical activity.

This raised concerns about why individuals with dynapenia tended to be obese. In our study, older adults with dynapenia had lower physical activity than those with sarcopenia, whereas those with presarcopenia had the highest physical activity among the four groups. This may partially explain why the dynapenic participants had much higher obesity parameters. In addition, some studies have explored whether the aging process increases lipid deposition in intermuscular and intramuscular regions [20, 21]. Fat infiltration into skeletal muscle may lead to muscle strength decline, metabolic dysregulation, and mobility limitation in older adults [20, 22–24]. In a C_2_C_12_ myotube model, insulin resistance was induced by treatment with palmitate. Intermuscular or intramyocellular lipids leading to the dysregulation of insulin signaling are characterized by abundant diacylglycerol (DAG) and ceramide accumulation [25, 26]. Fat infiltration into the muscle is associated with increased muscular expression of Perilipin2 with age in humans [27]. Evidence from these studies provides a possible mechanistic link among fat infiltration into the muscle, muscle function decline and the consequences of metabolic dysregulations. In our study, FMR followed by BFP seemed to be more relevant in representing the risk for low muscle strength than the other obesity parameters such as BMI, waist circumference or total fat mass.

In our study, we found that dynapenic older adults had a higher metabolic risk than sarcopenic individuals, whereas the individuals in the presarcopenic group had the lowest metabolic risk. Previously it was reported that aged adults with lower muscle mass had higher risk of metabolic syndrome, mainly in females [28]. Individuals with a low appendicular muscle mass to body weight ratio had a higher prevalence of metabolic syndrome in Korean population [29]. Kawamoto R et al. found that handgrip strength was inversely associated with metabolic syndrome among community-dwelling middle-aged and older persons [30]. Negative correlations between muscle mass, muscle strength and metabolic syndrome, independent of insulin resistance and central fat deposition were documented in Australian men aged 38-81 years [31]. In our study, we compared the impact of muscle mass and muscle function together for the risk of metabolic syndrome. We found that muscle function conferred a higher risk of metabolic syndrome independent of muscle mass. Previous studies reported that old adults with dynapenic obesity had a higher incidence of metabolic syndrome [32] or type 2 diabetes [33]. However, these studies defined dynapenia by low muscle strength only, without considering muscle mass. In addition, sarcopenia increased the risk of metabolic syndrome by 2.01-fold in middle-aged and older non-obese people was observed in a meta-analysis [34]. But in these analyses, obesity profiles were not considered either.

Participants with fall history tended to have lower handgrip strength and slower gait speed than non-fallers. However, it revealed no significant differences between fallers and non-fallers on body compositions, although the fallers tended to have higher obesity profile. This finding further supported that muscle quality played more important role in fall event rather than muscle quantity or any other body compositions.

In regard to the relationship between muscle mass, muscle function and fall risk, different studies used different parameters and cutoff values to examine this issue [35]. In addition, the clinical definitions of sarcopenia and dynapenia also varied [36]. Some studies had their own cutoff points derived from their own participants rather than from the consensus of the AWGS or EWGSOP. We found that low muscle function alone with or without low muscle mass seemed to be more relevant to higher fall risk, which is consistent with some other reports. Ida et al. found that in older diabetic men, low handgrip strength; but not sarcopenia, was correlated with fear of falling [37]. Tanimoto et al. found that subjects with low muscle strength or performance had a slightly higher fall risk than those with reduced muscle mass in women [38]. In our study, dynapenic older adults had a higher fall risk than sarcopenic individuals even after adjusting for possible confounding factors. We also found that presarcopenic older people tended to have lower fall risk. Therefore, for fall risk, muscle function rather than muscle mass matters.

Low muscle mass alone may not be a risk factor for functional decline in the absence of obesity [36]. Scott D and colleagues showed that dynapenic obesity, but not sarcopenic obesity, was expectative of an increased fall risk scores among older adults in a five-year prospective cohort study [39]. In our study, dynapenic older adults tended to be more obese than sarcopenic individuals, and this finding may be partially linked to the result of a higher fall risk among the individuals with dynapenia.

The older people in the robust and dynapenia groups have normal muscle mass with different muscle function. Their fat mass, FMR, risks of fall and metabolic syndrome were different. In contrast, low muscle mass defined both presarcopenia and sarcopenia groups, however, the difference in muscle function was related to their distinct patterns in fat mass, FMR, risks of fall and metabolic syndrome. Taken together, these results showed muscle function seems to be more relevant to the risk of metabolic syndrome and reported fall events, irrespectively of muscle mass itself. Obesity either dynapenic (with normal muscle mass) or sarcopenic (with reduced muscle mass) is associated with reduced muscle function.

The new EWGSOP screening protocol on sarcopenia already considers muscle strength as the first step for screening [40]; however, this revision did not emphasize that the participants with dynapenia have higher risk of clinical adverse events than the other groups**.** In fact, body fat measurements, the most distinguishing characteristic for muscle function decline, were rarely explored in previous reports. In addition, FMR seems to be a more suitable parameter in estimating low muscle strength and low physical performance.

Some limitations of this study need to be mentioned. The fall risk assessment was obtained by participants’ self-report, and recall bias could not be avoided. All participants were enrolled from the urban community, and they were relative healthy and ambulatory. This study may not be applied to the elderly population with severe illness leading to the limited generalizability to other population. Although we have adjusted multiple covariates in the regression model; nevertheless, cognitive function and nutritional status were not available in our study bring about the restriction to minimize the possible confounding effects. In addition, this was a cross-sectional study, causal relationships between different stages of sarcopenia and their impact on clinical risks can not be inferred. Prospective studies to elucidate these relationships are warranted.

## CONCLUSIONS

Community-dwelling older people with dynapenia were more obese and had a higher risk of fall and metabolic syndrome than sarcopenia individuals. In contrast, presarcopenia older adults were less obese and had a lower risk of metabolic syndrome. Muscle function was more important than muscle mass on fall risk. Increased adiposity with or without reduced muscle mass was related to muscle function decline.

## MATERIALS AND METHODS

### Study participants

This is a cross-section observational study. From March 2015 to July 2017, adults who lived in the urban community with age of 65 and older were eligible when they attended an annual routine health check-up program at Tri-Service General Hospital (TSGH). Participants who had ever experienced chest pain or bone pain while doing exercise, had problems of cognitive impairment or congestive heart failure, were currently under regular hemodialysis, or had a malignancy that required medical therapy, or had pacemaker implantation were excluded. They could walk by themselves from community to our medical center and they are relatively healthy in general. All old people were invited unless they refuse to join this study. All participants were requested to do physical exercise such as holding hands and walking at usual pace. If they could not do these physical exercises they were supposed not eligible to participate in this study. At first, trained investigator will screen the subjects by medical record reviewing to find out whether they have the exclusion conditions mentioned above. If they were eligible for this study, the trained investigator will invite them to participate and provide inform consent.

Information regarding the demographic profile of the participants, their overall health condition, and physical activities were obtained through a structured questionnaire. Twenty milliliters of venous blood was collected after overnight fasting. Participants’ age, health behaviors such as cigarette smoking and alcohol drinking were ascertained from a personal identification card and by self-report. Alcohol consumption was defined as alcohol beverage drinking at least once every week currently and was dichotomized. Positive smoking status was indicated as ever smoking in life and was treated as categorical variable. The presence of hypertension was defined as an average blood pressure with 140/90 mmHg or higher, based on a physician's diagnosis that was self-reported or based on the use of medicines for blood pressure control. Diabetes mellitus was defined as fasting plasma glucose ≥126 mg/dl, self-report of a doctor's diagnosis, or current use of anti-diabetic medications (including oral hypoglycemic agents or insulin injection). Medical history including stroke, heart diseases, chronic lung diseases, arthritis, osteoporosis, use of antipsychotic agents or sedative agents and so forth were obtained by self-report. International Physical Activity Questionnaire Short Form (IPAQ) was used to assess participant’s physical activities [41]. Participants were recorded as having a positive recent fall history if they had experienced a fall event in the past one year. All participants provided written informed consent prior to participation. The protocol was approved by the Institutional Review Board of TSGH.

### Measurement of body composition

Body weight was measured by a digital scale to the nearest 0.01kg; and body height was checked with a stadiometer to the nearest 0.1 cm. Body weight in kilograms divided by the square of body height in meters was indicated as body mass index (BMI). The waist circumference (WC) was measured at the mid-level between the iliac crest and the lower border of the 12^th^ rib while participant was standing with feet 25–30 cm apart. Bioelectronics impedance analyzer (BIA) was used to measure appendicular skeletal muscle (ASM) (InBody 720, Biospace, Seoul, South Korea). We used body height in meter to adjust ASM, i.e., ASM/ht^2^, to define the skeletal muscle mass index (SMI). We also measured body fat mass and body fat percentage (BFP) by BIA. The fat to muscle ratio (FMR) was calculated as body fat mass divided by body muscle mass. The cutoff value of low SMI was 7.0 kg/m^2^ in men and 5.7 kg/m^2^ in women according to the consensus from the Asia Working Group of Sarcopenia (AWGS) in 2014 [42].

### Functional performance measurement

Measurement of the average value of the dominant hand’s grip strength was conducted three times by an analogue isometric dynamometer (Exacta™ Hydraulic Hand Dynamometer; North Coast Medical Inc., Gilroy, CA). Six-meter distance walking time was measured for all participants, where they were asked to go as usual or habitual walk speed. Distance divided by walking time expressed in m/s was indicated as gait speed. Based on the sarcopenia definition by AWGS in 2014, men with handgrip strength below 26 kg was defined as low handgrip strength; whereas the cutoff value of low handgrip strength for women was lower than 18 kg. The gait speed ≤ 0.8 m/sec in both genders is the cutoff point for low gait speed [42].

### Definition of robust, dynapenia, presarcopenia and sarcopenia

Robust was defined as having normal SMI, normal handgrip strength and normal gait speed. Participants with normal SMI but had low handgrip strength and/or low gait speed were categorized as dynapenia. Presarcopenia was defined as low SMI with normal handgrip strength and normal gait speed; sarcopenia was indicated by low SMI with either low handgrip strength or low gait speed or both, according to the consensus of European Working Group on Sarcopenia in Older People (EWGSOP) in 2010 [8]. The cutoff values of low SMI, low handgrip strength and low gait speed were consistent with the consensus of AWGS in 2014 [42].

### Definition of metabolic syndrome

We used the National Cholesterol Education Program (NECP) expert panel on the Detection, Evaluation, and Treatment of High Blood Cholesterol in Adults (Adult Treatment Panel III) guideline [43] to define metabolic syndrome. Modified criteria of abdominal obesity for Asian people were cited from the International Diabetes Federation [44]. Metabolic syndrome was defined as having three or more of the following conditions: (1) elevated blood pressure, systolic blood pressure ≥130 mmHg or diastolic blood pressure ≥85 mmHg, or with treatment of hypertension; (2) impaired fasting glucose, fasting glucose ≥100 mg/dl, or with treatment of diabetes mellitus; (3) hypertriglyceridemia, fasting serum triglyceride ≥150 mg/dl, or with treatment for hypertriglyceridemia; (4) low level of high-density lipoprotein, serum high-density lipoprotein <40 mg/dl in men or <50 mg/dl in women; and (5) central obesity, waist circumference ≥90 cm in men or ≥80 cm in women.

### Statistical analysis

Participants’ characteristics with continuous variables were represented as the mean± standard deviation; whereas the categorical data were expressed as numbers with percentages. ANOVA was conducted to examine the differences of continuous variables among robust, dynapenia, presarcopenia and sarcopenia groups. Student-t test was performed to examine the differences of muscle strength, body compositions between faller and non-faller groups. We used multiple logistic regression analysis to examine the risk of metabolic syndrome and fall event among those with dynapenia, presarcopenia and sarcopenia. In the risk analysis of metabolic syndrome, we adjusted multiple covariates by an extended-model approach: Model 1 = age and gender; Model 2 = Model 1 plus behaviors of personal health (cigarette smoking and current alcohol consumption), physical activities, uric acid, stroke and coronary artery disease. Different covariate adjustments were also applied for fall risk estimation: Model 1 = age and gender; Model 2 = Model 1 plus behaviors of personal health (cigarette smoking and current alcohol drinking), metabolic syndrome, physical activities, osteoporosis, arthritis, and use of antipsychotic agents and sedative agents. By calculating the area under the curve (AUC) in receiver operating characteristic (ROC) analysis, we assessed the diagnostic performance of different obese parameters on low handgrip strength and low gait speed. We used Statistics Package for Social Science version 16.0 software (SPSS, Inc., Chicago, IL) to conduct all analyses.

## Supplementary Material

Supplementary Figure 1
