# Supplementary material for: Impact of adiposity on muscle function and clinical events among elders with dynapenia, presarcopenia and sarcopenia: a community-based cross-sectional study
Source: Aging (Albany NY). 2021 Feb 26;13(5):7247–58. doi: 10.18632/aging.202581 (PMC7993704; doi:10.18632/aging.202581)
Supplement: Supplementary Figure 1 [file aging-13-202581-s001.pdf]

SUPPLEMENTARY FIGURE

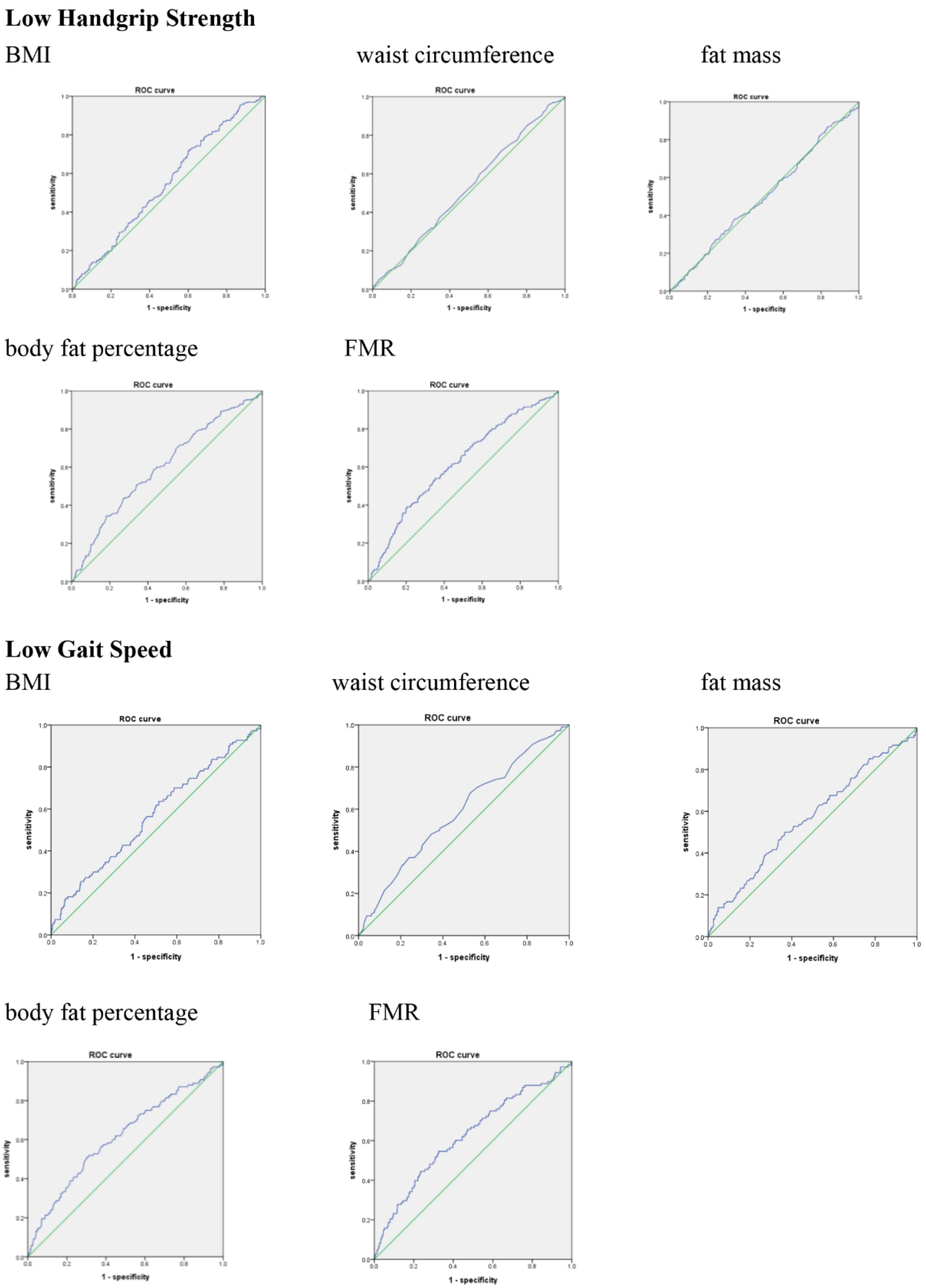

Supplementary Figure 1. The receiver operating characteristics (ROC) curve to estimate the area under the curve (AUC) between different obesity parameter and low muscle function. In estimating the risk for low handgrip strength, FMR had the best diagnostic performance among these obese parameters (AUC= 0.617, 95% CI = 0.573-0.661). In the prediction of low gait speed, the best diagnostic performance was still FMR (AUC= 0.622, 95% CI = 0.563-0.682) than by the other obese parameters. BMI, body mass index; CI, confidence interval; FMR, fat to muscle ratio.
